# Role of Pharmacogenetics in Improving the Safety of Psychiatric Care by Predicting the Potential Risks of Mania in CYP2D6 Poor Metabolizers Diagnosed With Bipolar Disorder

**DOI:** 10.1097/MD.0000000000002473

**Published:** 2016-02-12

**Authors:** Santiago Sánchez-Iglesias, Virginia García-Solaesa, Belén García-Berrocal, Almudena Sanchez-Martín, Carolina Lorenzo-Romo, Tomás Martín-Pinto, Andrea Gaedigk, José Manuel González-Buitrago, María Isidoro-García

**Affiliations:** From the Servicio de Psiquiatría, Hospital Universitario de Salamanca (SS-I, CL-R, TM-P); Instituto Biosanitario de Salamanca, IBSAL (VG-S, BG-B, AS-M, JML-R, MI-G); Servicio de Bioquímica Clínica, Hospital Universitario de Salamanca (BG-B, JMG-B, MI-G); Servicio de Farmacia, Hospital Universitario de Salamanca, Spain (AS-M); Division of Clinical Pharmacology, Toxicology and Therapeutic Innovation, Children's Mercy Hospital (AG); Department of Pediatrics, School of Medicine, University of Missouri-Kansas City, Kansas City, MO, USA (AG); and Departamento de Medicina, Universidad de Salamanca, Spain (MI-G).

## Abstract

One of the main concerns in psychiatric care is safety related to drug management. Pharmacogenetics provides an important tool to assess causes that may have contributed the adverse events during psychiatric therapy. This study illustrates the potential of pharmacogenetics to identify those patients for which pharmacogenetic-guided therapy could be appropriate. It aimed to investigate CYP2D6 genotype in our psychiatric population to assess the value of introducing pharmacogenetics as a primary improvement for predicting side effects.

A broad series of 224 psychiatric patients comprising psychotic disorders, depressive disturbances, bipolar disorders, and anxiety disorders was included. The patients were genotyped with the AmpliChip CYP450 Test to analyzing 33 allelic variants of the CYP2D6 gene.

All bipolar patients with poor metabolizer status showed maniac switching when CYP2D6 substrates such as selective serotonin reuptake inhibitors were prescribed. No specific patterns were identified for adverse events for other disorders.

We propose to utilize pharmacogenetic testing as an intervention to aid in the identification of patients who are at risk of developing affective switching in bipolar disorder treated with selective serotonin reuptake inhibitors, CYP2D6 substrates, and inhibitors.

## INTRODUCTION

The use of pharmacogenetics in psychiatry is increasingly implemented into clinical practice although there is still limited information supporting their use.^1^ Pharmacogenetic tests are available for the practice of psychiatry to gather genetic information aimed at personalized medicine.^2^ Approximately, 25% of currently used drugs are metabolized by CYP2D6.^3–6^ To date over 100 allelic variants have been defined for *CYP2D6*, (http://www.cypalleles.ki.se/). Updated allele frequencies can be found in the Clinical Pharmacogenetics implementation Consortium (CPIC) guidelines.^7^ Extensive metabolizers (EMs) are patients with at least 1 fully functional allele. Ultrarapid metabolizers (UMs) show increased metabolic activity. The term intermediate metabolizer (IM) refers to individuals with 1 reduced activity allele and 1 null allele. Patients with reduced activity may be at greater risk of adverse events or may bioactivate prodrugs in a less efficient way.^4,8^ Finally, poor metabolizers (PMs) with 2 nonfunctional alleles are more likely to experience dose-related adverse drug reactions compared to EM patients.^9–12^ Currently, used antidepressant drugs such as paroxetine or fluoxetine are potent inhibitors of CYP2D6 and their use may alter a patient's phenotype from EM to PM in a process called pheno-copying^13,14^ what constitute an added problem since multiple drugs could have a potential inhibitory effect on CYP2D6. Our study aimed to investigate *CYP2D6* genotype in our psychiatric population to assess the value of introducing pharmacogenetics as a primary improvement for predicting side effects.

## MATERIAL AND METHODS

### Study Population

A total of 224 patients diagnosed with psychotic disorders, depressive disturbances, bipolar disorders (BPD), and anxiety disorders were included. Patients started a medium-term treatment (12–24 months) with antidepressants or antipsychotics while hospitalized in a short-term unit (54%), and patients on an outpatient regime (mental health unit) who started pharmacological treatment with antidepressants or antipsychotics for which a prolonged duration (5 years) of drug therapy was foreseen (46%) were included. The clinical variables collected by physicians of the psychiatry service included, among others, age, gender, ethnicity, diagnosis, medical comorbidities, type number and duration of psychiatric treatment, clinical global impression, and adverse drug reaction (UKU scale); physicians were blinded to genotype results. Regarding ethical issues, the Institutional Review Board of the University Hospital of Salamanca approved the study; all subjects gave written informed consent to the genetic testing. Aspects related to privacy concerns, protection of participants, and physical well-being were addressed.

### Planing the Intervention

The intervention planned here is based on the pharmacogenetics analysis of our patients by genotyping studies. P450 metabolizer enzymes are considered more likely to influence drug side effects. The intervention consisted in genotyping CYP2D6 gene in the psychiatric patients. The AmpliChip CYP450 Test (Roche Molecular Systems, In Indianapolis) approved by the Food and Drug Administration in 2005,^15^ was chosen because analyses 33 allelic variants of *CYP2D6* including the most common variants observed across ethnicities. The majority of sequence variations detected are single nucleotide polymorphisms. The AmpliChip also detects the presence of a number of *CYP2D6* gene duplications and the *CYP2D6*^*∗*^*5* gene deletion. Simultaneously, 2 additional single nucleotide polymorphisms are interrogated to determine the presence of *CYP2C19*^*∗*^*2 and*^*∗*^*3*. The analysis was prescribed by the psychiatrists after collecting the previous information and performed in the pharmacogenetics laboratory. DNA was extracted from 1 mL of whole blood collected into EDTA-containing vacutainers with the MagnaPure Compact system (Roche Applied Science, Mannheim, Germany). AmpliChip CPY450 Test was performed as recommended by the manufacturer.^16^

### Methods of Evaluation

The intervention was developed in all patients included in the study. To assess how well the intervention was implemented clinical and laboratory supervision was established during all the study assigning a responsible for each task. It is expected that the intervention provides information that could be useful to predict adverse effects in these patients. To assure data quality, strict control and appropriate scales were used to data collection. In addition, the genotyping was performed following the directives of the European Molecular Genetics Management Network^17^ for DNA handling, with the requisite controls. The application of quality norms followed the UNE-EN-ISO 15189:2007 Normative in the Accredited Section of Molecular Genetics and Pharmacogenetics of the Clinical Biochemistry Service of the University Hospital in Salamanca. The normative included training and qualification of personal, preanalytical, analytical and postanalytical control, blinding, repeating measurements, and internal and external validity.

### Data Analyses

Qualified statistical analysis was performed with SPSS v.17 (IBM, Chicago, IL, EEUU). For the analysis of the qualitative variables the Chi-square test was employed. In order to compare the qualitative and quantitative variables, ANOVA was performed after analysis of the homogeneity of variance. Determination of allele and genotype frequencies was accomplished with the Shesis platform.^18^

## RESULTS

Diagnostic categories observed in our cohort were schizophrenia and other psychotic disorders (F20–29) (n = 76, 33.9%); depressive disturbances (F32–34) (n = 72, 32.1%), BPD (F30–31) (n = 45, 20.1%), anxiety disorders (F40–43) (n = 16, 7.1%), and others psychiatric disorders (n = 15, 6.7%). The mean age of the patients was 45.3 years (SD 15.4) and 56.7% were women. The ethnicity of the subjects was predominantly Caucasian (95.8%).

The intervention provided information about genotype distribution of *CYP2D6* gene and the predicted phenotype. Of the 224 patients, 4.5% had *CYP2D6* genotypes predicting UM, 9.8% IM, and 6.3% PM metabolizer phenotype. The most frequently observed genotypes were *CYP2D6*^*∗*^*1/*^*∗*^*2xN* for UM group, ^*∗*^*4/*^*∗*^*41* for IM group, and ^*∗*^*4/*^*∗*^*4* in the PM group. Allele and genotype frequencies for the study cohort are shown in Tables 1 and 2. We did not find any correlations between metabolizer status and the studied disorders. For 6 patients the AmpliChip CPY450 Test returned “no-call” results that were solved by other methods.^19,20^ A total of 90.1% of the patients received at least 1 drug metabolized by CYP2D6. In the bipolar group 77.8% of patients (35) were diagnosed with bipolar disorder type I (BPI) and 22.3% (10 patients) were bipolar disorder type II (BPII). In the total group of BP patients, 3 patients were predicted to be PM (6.7%) and 2 patients to be IM (4.4%). Interestingly, 4 of these 5 patients (80%) experienced a maniac switching (ie, an episode defined as a sudden transition from a mood episode to another episode of the opposite polarity), when a simultaneous substrate and inhibitor of CYP2D6 was prescribed. There were no associations between adverse events reported by patients treated for depressive, psychotic or anxiety disorders, and CYP2D6 genotype or predicted phenotype status. The most relevant outcome of the intervention was the identification that all bipolar patients with *CYP2D6* PM genotypes experienced these episodes when drugs that are simultaneous substrate and inhibitors of CYP2D6 were prescribed.

**TABLE 1:**
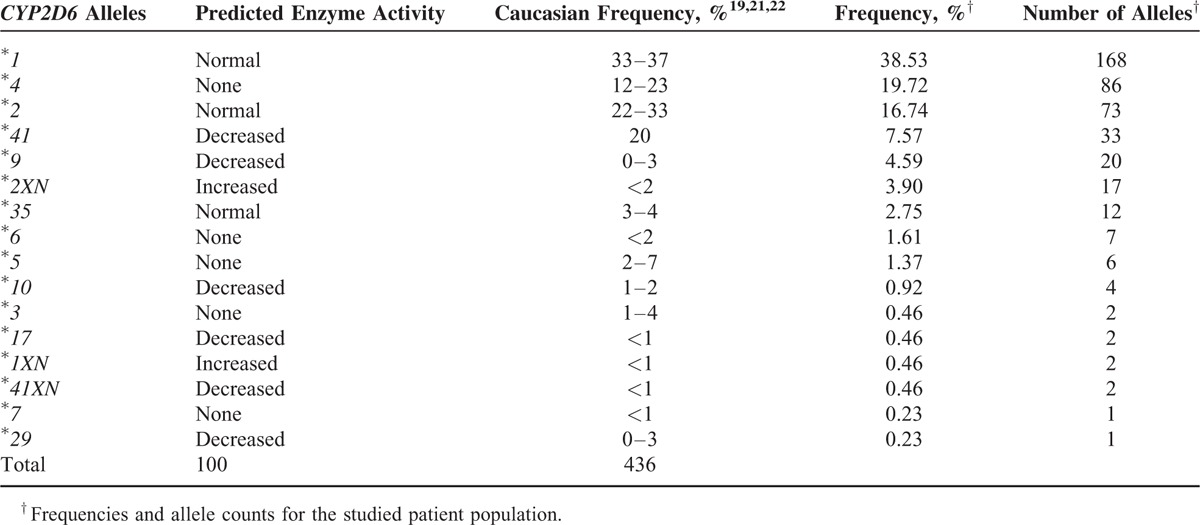
Allelic Frequencies for CYP2D6 Gene in the Population Studied and in Normal Population^19,21,22^

**TABLE 2:**
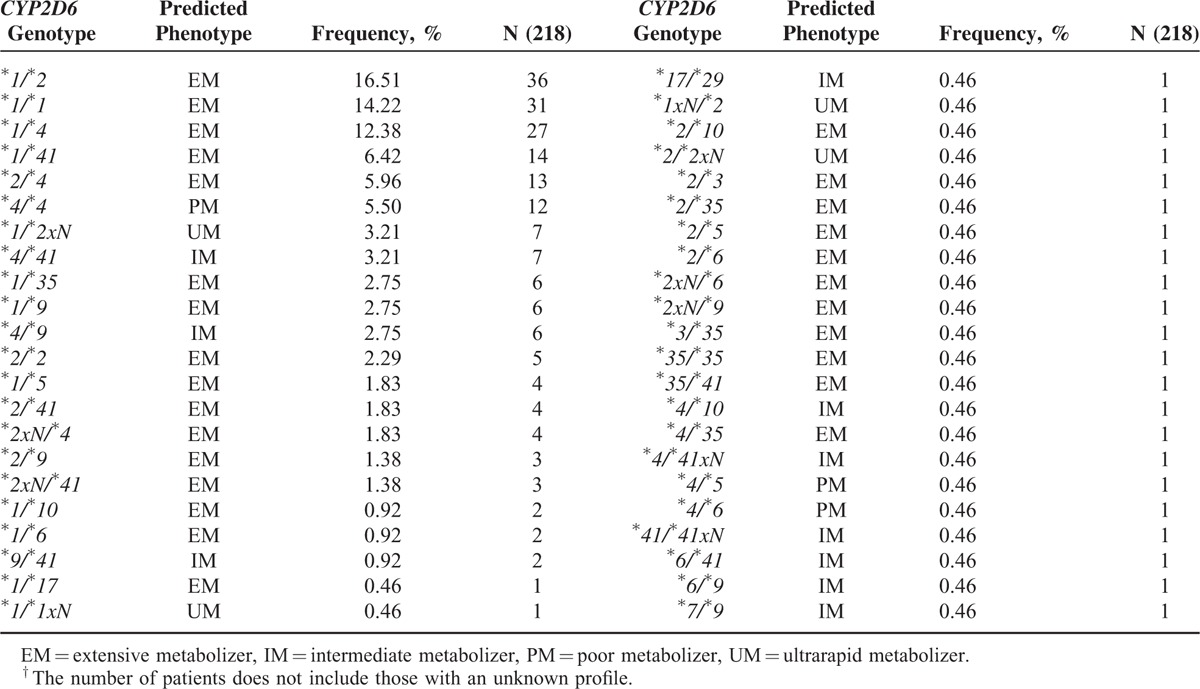
Distribution of Genotype Frequencies of CYP2D6 Gene in Our Population^†^

## DISCUSSION

Our study aimed to identify the utility of CYP450 genotyping for predicting side effects in psychiatric patients. Clinical relevance of pharmacogenetic testing is supported by the high proportion of patients with genotypes predicting either intermediate or PM status of the CYP2D6 enzyme system that constitutes the main metabolic pathway for a large number of the psychiatric drugs in current use.

Furthermore, about 80% of patients with EM-predicting genotypes received drugs that inhibit CYP2D6. These patients will likely have reduced CYP2D6 capacity or even convert to the PM phenotype.^23^ The shift from EM (or IM) to PM status often appears after treatment with selective serotonin reuptake inhibitors (SSRIs), which needs to be considered in clinical practice. This is especially important when SSRIs are used in combination with other drugs that are metabolized via the CYP2D6 pathway.^24^ Drugs that are simultaneous substrates and inhibitors of CYP2D6 can contribute to a substantial reduction in their own metabolism and thereby lead to increase their plasma levels. It should be noted that more than 200 drugs could have a potential inhibitory effect on CYP2D6.^25^

Patients suffering from bipolar disorders may present with a maniac (or hypomaniac) episode or with depressive episodes. In the latter case, switching is a major risk during the treatment of bipolar depression. Our most striking observation was that all bipolar patients with PM status experienced a maniac episode after introducing a substrate of CYP2D6 such as paroxetine or fluoxetine to their treatment regimes. In PM patients, an exaggerated pharmacological response or toxicity would be expected for drug deactivated by CYP2D6. In this sense, pharmacokinetic modifications of different psychiatric drugs, according to the metabolizer status, have been previously reported. The individualizing drug therapy has been discussed in the literature^26,27^ and the CPIC recommends reducing the dose for SSRIs metabolized by CYP2D6 for PMs.^7^ A list of our clinical practice in patients with genetically impaired CYP2D6 metabolism is shown in Table 3.

**TABLE 3:**
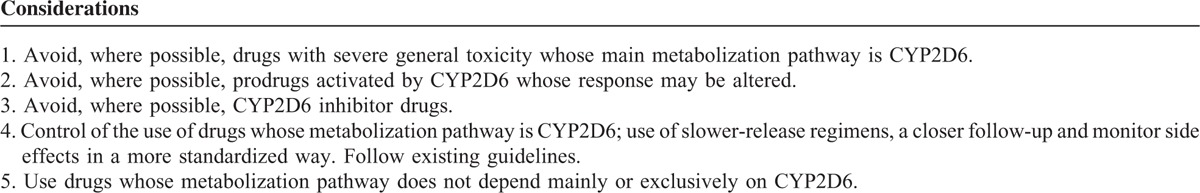
List of Our Clinical Practice in Patients With Genetically Impaired CYP2D6 Metabolism

Several neurobiological factors have been described associated with mood episode switches^28^ and the propensity to mood switches in bipolar patients have been described subjected to individual differences.^29^ The treatment emergent affective switch has been broadly described: antidepressants have been associated with an increased risk of inducing mania^30,31^ and may increase maniac symptom severity^32^ in bipolar patients. However, the literature is controversial, that is, in some clinical trials the number of maniac switches was similar between placebo and antidepressant groups.^33^ Pharmacogenetics could help to explain this controversy since the metabolizer status of the patient likely impacts the response to drugs used in clinical trials.

We would also like to emphasize that there is uncertainty regarding the potential harm or benefit associated to antidepressants used in BPD. Recent data suggest absent or very weak efficacy data for paroxetine in bipolar treatment.^34^ Although, European guidelines exert a more allowing attitude, US-guidelines do not recommend antidepressants in bipolar depression, unless depression is severe. As previously commented, many factors have been involved in antidepressant induced affective side-effects, including comorbidities, a history of mania, early beginning, psychotic features, or even the type of BPD. BPII subtype has been associated with low switch rates. This is in accordance to our results because all patients with maniac switch were BPI. Our aim was to assess CYP2D6 genotype as an intervention for analyzing the treatment of bipolar patients. Our findings suggest that a patient's CYP2D6 metabolic profile contributes to maniac switching in BPI patients.

Personalizing treatment regimens allow the physician to avoid certain drug combinations in at-risk patients, that is, those with reduced or no appreciable CYP2D6 activity. Personalized medicine research is a more person-centered and thus eventually more humanistic diagnostic and therapeutic approach.^35^ Pharmacogenetic testing allows specialists to interpret genotyping results within the context of the clinical history providing guidance for drug selection and dosage. This highlights the importance for providers to become more aware of the clinical relevance of pharmacogenomic tests.^36^ As an example, The CPIC has published guidelines for *CYP2D6* genotypes and dosing of tricyclic antidepressants^2,37,38^ and SSRIs.^39^ A selection of drugs that are commonly used in psychiatry with pharmacogenomics information in their label per the U.S. Food and Drug Administration is provided in Table 4. Table 5 provides a list of drugs, whose metabolism does not depend on CYP2D6 or CYP2D6 is not the main metabolizer.

**TABLE 4:**
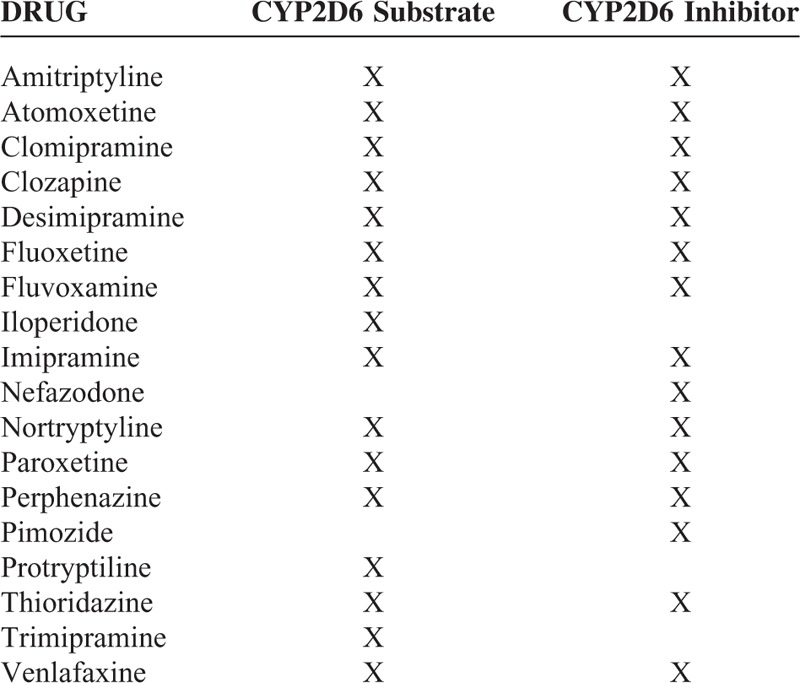
This Table Provides a Selection of Drugs Commonly Used in Psychiatric Practice, Which Have Pharmacogenetic Information in Their Label per the U.S. Food and Drug Administration

**TABLE 5:**
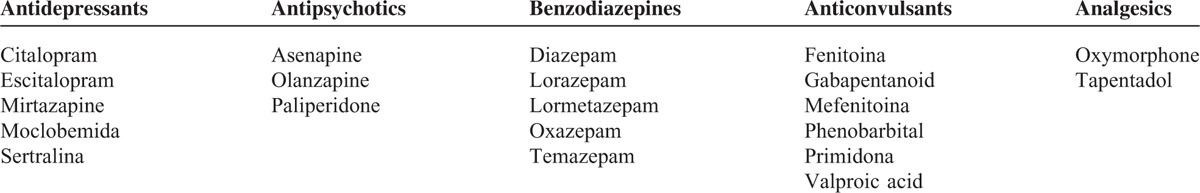
Metabolism of the Drugs Shown Does Not Depend on the CYP2D6 or CYP2D6 Is Not the Main Pathway

One of the difficulties in implementing the intervention is the cost of genotyping; however, the costs of the molecular methodology are decreasing. In addition, the genotyping is performed once in life and the information results useful not only regarding the actual medication but also for all drugs metabolized by these enzymes. One has to be aware, however, that genetic variation in other genes, for example, drug transporters or drug targets, as well as other factors likely contribute to individual drug response.^40^ We also acknowledge limitations of this study. For example, the AmpliChip P450 Test does not capture all known allelic variants for *CYP2D6* and other techniques are needed in these cases. Although the AmpliChip P450 Test also provides *CYP2C19* genotype this test does not include potentially relevant *CYP2C19* alleles such as *CYP2C19*^*∗*^*4* and ^*∗*^*17*. Therefore, data of this gene are not shown in this report.

## WHAT IS NEW AND CONCLUSIONS

Preemptive genotype-based phenotype (metabolizer status) prediction could be a useful clinical tool for the individualization of the treatment while retrospective genotyping may be informative regarding adverse events. Subjects at the extreme distributions of CYP2D6 activity are those at highest risk. Some commonly used drugs CYP2D6 inhibitors can dramatically lower a patient's enzymatic activity and thereby shift the patient metabolic capacity toward the low end of the activity distribution. In our experience, *CYP2D6* genotype information has been very useful in the psychiatric setting especially in BPD. Our study provides new insights that are also valuable for future studies in the field. We demonstrate the utility of pharmacogenetic testing to explain severe adverse events in CYP2D6 PMs diagnosed with BPD. Our observations further support the implementation of preemptive pharmacogenetic testing as it can provide substantial benefits increasing drug safety in these patients. Based on our observations, we propose to consider pharmacogenetics as a valuable intervention to help identifying patients at risk to develop affective switching in BPD treated with SSRIs or other CYP2D6 substrates and inhibitors.
